# New Advances in Cement and Concrete Research

**DOI:** 10.3390/ma16114162

**Published:** 2023-06-02

**Authors:** Gun Kim

**Affiliations:** Department of Urban and Environmental Engineering, Ulsan National Institute of Science and Technology (UNIST), UNIST-gil 50, Ulju-gun, Ulsan 44919, Republic of Korea; gunkim@unist.ac.kr

The Special Issue (SI), “New Advances in Cement and Concrete Research”, highlights the latest breakthroughs in cement and concrete research. These advances include novel strategies for reducing CO_2_ emissions in cement production, carbon sequestration into cement, the identification of new agents for enhancing durability, the development of ultra-high-performance concrete (UHPC) with compressive strengths exceeding 170 MPa, radiation shielding, and the enhancement of the durability of concrete in extreme environments by employing mix designs.

Wojtacha-Rychter et al. [1] explored the environmental and economic advantages of utilizing refuse-derived fuels (RDF) and sewage sludge (SS) in cement kilns. Their findings revealed that substituting 90% of coal with RDFs can save up to 28.6 metric tons per hour of coal, while increasing the SS fraction in total heat consumption by 6% can decrease emissions by 17 kg of CO_2_ per metric ton of clinker. This approach has the potential to significantly reduce the carbon footprint of cement production, a major source of greenhouse gas emissions. Sim et al. [2] introduced a carbon sequestration technology that employs concrete slurry water (CSW) generated during concrete production as a new CO_2_ sink to lower CO_2_ emissions from the cement industry. Their research demonstrated that complete carbonation can be achieved within 10 min at specific CSW ratios (5–25%), with the ability to reduce CO_2_ emissions from the cement industry.

Amin et al. [3,4] investigated radiation attenuation in concrete using artificial neural networks (ANN) and gene expression programming (GEP) to quantitatively predict the degree of shielding. The results effectively demonstrated the relationship between concrete geometry and shielding efficiency. Lee et al. [5] developed an ANN-integrated numerical model to inversely estimate moisture diffusion. This study demonstrated the potential of machine learning and provided a basis for quantifying the effect of moisture distribution on the durability of in-service concrete structures.

Alternative materials and innovative mix designs are being explored to improve concrete performance. Kumar et al. [6] assessed the effects of substituting rice husk ash and soap solutions for cement on the permeability and carbonation resistance of concrete. Arbili et al. [7] examined the use of iron ore tailings and their effect on concrete strength and durability, while Al-Kharabsheh et al. [8] employed wood ash as a partial substitute for cement and studied its effect on concrete strength and durability. Xie et al. [9] proposed a novel mixture design for UHPC based on a modified Fuller distribution model. The UHPC matrices optimized by the proposed model, which replaced granulated blast furnace slag (GBFS) with quartz powder (QP), exhibited excellent performances: a slump flow of 740 mm, compressive strength of 175.6 MPa, tensile strength of 9.7 MPa, and flexural strength of 22.8 MPa.

Niu et al. [10] examined the fatigue life of UHPC using J-integral and digital image correlation (DIC) methods. They evaluated the patterns of fatigue crack propagation in UHPC with varying steel-fiber lengths under cyclic loading, and they ascertained the relationship between the critical crack length and fiber length. Choi et al. [11] developed a novel cement-repair material tailored for cold weather conditions. Specifically, they designed nitrite/nitrate-based anti-freezing agents to promote hydration and strength development. The balance between the strength and dosage of these antifreeze agents was determined in order to meet the requirements for repairing and maintaining cementitious materials in cold regions, such as Hokkaido, Japan.

The findings presented in this SI are useful in the ongoing efforts to enhance the sustainability, durability, and performance of cement and concrete products. Moreover, such advances may help address the challenges of evaluating the sustainability of concrete materials and climate change factors that could influence the design of such materials.
